# Prevalence of Depression Among People With Dry Eye Disease: Empirical Analysis From the Southern Region of Saudi Arabia

**DOI:** 10.7759/cureus.39253

**Published:** 2023-05-20

**Authors:** Abdulrahman Alamri, Sultan Bakri, Reemah Alqahtani, Lama Al Hadi, Abdulkhaliq H Asiri, Najla Alshehri, Amal Alqarni, Faisal Alamri, Mohammed Althwabi, Kholoud Alrobaie

**Affiliations:** 1 Ophthalmology, King Khalid University, Abha, SAU; 2 Ophthalmology, Imam Abdulrahman Bin Faisal University, Dammam, SAU; 3 College of Medicine, King Khalid University, Abha, SAU

**Keywords:** prevalence, saudi arabia, empirical analysis, dry eye disease, depression

## Abstract

Background

The number of people suffering from depression has increased in recent times. In the Aseer region alone, 3.8% of the population suffers from depression, and one of the causes is believed to be dry eye disease (DED).

Objectives

This research aims to investigate the relationship between depression and dry eye disease among people living in Saudi Arabia’s Aseer region.

Methodology

The study is cross-sectional, and data was collected from 401 participants living in Aseer, Saudi Arabia. Data was collected with the help of a well-structured questionnaire, and results are extracted by analyzing the model using SPSS.

Findings

The study discovered a positive and significant relationship between dry eye disease and depression. A total of 36.7% of the participants had dry eye symptoms and 23.7% were diagnosed with depression, stress, or anxiety.

Conclusion

Our study concludes that patients suffering from dry eye disease are more likely to get depressed because, according to our analysis, there is a correlation between dry eye disease and depression. Dry eye disease is a condition that is not only affecting the elderly but the young alike. Saudi Arabia’s healthcare authority should create awareness about this health issue with the help of seminars, print, and social media.

## Introduction

Dry eye disease (DED) has become a major health issue in ophthalmology. DED is a multifunctional disorder of tears and ocular surface caused by uneasiness, visual disturbance, and tear film variability with potential injury to the ocular surface. It is accompanied by increased osmolarity of the tear film and tenderness of the ocular surface. About 5%-50% of the population in any area suffers from DED [1-3]. Signs of dry eye disease include dryness, foreign body feeling, pain, inflammation, etc.

The symptoms can remain for a long time, and this condition substantially negatively influences a patient’s daily routine. DED can be caused by multiple issues such as collagen syndrome, malnutrition, implantation disease, medications, etc. The incidence rate of DED is growing in developed countries, even though the etiology of this disorder is multifactorial and remains unidentified.

The relationship between DED and psychiatric disorders has been gaining attention in recent years. It is stated that major depression is higher in patients with DED, and multiple researchers have linked depression with dry eye disease [4]. Still, previous studies have restrictions, such as a lack of close inspection of DED or mood symptoms. Some studies did not apply internationally authenticated measurement scales. Furthermore, no studies have monitored the mood of patients with DED. So, to the best of our knowledge, no such kind of study has been conducted in Saudi Arabia. This highlights the importance of conducting a study that unravels the link between DED and depression. While a recent survey in Saudi Arabia showed a 42% risk of depression among patients with dry eye disease [5], after an increase in incidences of dry eye disease and depression, this study tried to investigate the relationship between depression and dry eye disease in a larger sample from Aseer region, Saudi Arabia to try to determine if depression is caused by dry eye disease.

## Materials and methods

Study design

The current study is cross-sectional, and data was collected with the help of a questionnaire. The questionnaire was translated into Arabic for tranquil approachability to the targeted population [6]. A psychiatrist also verified the questions to check the authenticity of questionnaires in the context of depressive symptoms. The target population is Saudi Arabia. This study included participants aged 15 and older who were either previously diagnosed with dry eyes or had a dry eye disease. 

For exclusion criteria, this study excluded all persons who were previously diagnosed with depression or suffering from any other psychiatric illness, have more than one comorbid health condition such as heart disease, diabetes, asthma, hypertension, etc., or had a recent ocular procedure done in less than a year including laser-assisted in-situ keratomileusis (LASIK), crosslinking, or corneal transplantation. Other data includes biographical information such as age, gender, province/city, eye conditions, eye procedures, daily screen time, and contact lens use. Every participant gave informed consent. Moreover, they were guaranteed that all the information they gave was only analyzed for scientific study reasons and would be kept confidential.

Data collection

We conducted a pilot study on about 20 participants in order to ensure the questionnaire will give more accurate results. The pilot study also helped us to verify the language correctness and validity of questions. 

In preparing the questionnaire, we ensured it had the capacity to assess numerous variables starting with demographic variables such as occupational status, educational level, marital status, gender, and age. We used a Depression Anxiety Stress Scales-21 (DASS-21) that was validated in Arabic. A DASS-21 questionnaire was utilized to determine the severity of anxiety, depression, and stress. Part of the questions in the questionnaire assessed dry eye disease by utilizing the Ocular Surface Disease Index (OSDI) questionnaire. 

We formed various groups of participants by classifying them according to their years. These are < 18, 18-45, 45-55, and ≥50. On education, we classified them into High school or below, Bachelor's or diploma, and Master's or doctorate. On marital status, participants were categorized into single or married. On employment status, the groups were based on the following criterion: unemployed, employed, and retired. Participants were asked whether they had been previously diagnosed with dry eye and the choices were yes and no. 

In the other part of the questionnaire, the participants answered some days only, most days, not at all, and almost every day. They were asked questions on whether or not they have suffered from refractive error, physical ailments, other eye diseases, eye redness, light sensitivity, anxiety, stress, depression, and if they have ever felt the presence of a foreign body in their eyes. They were asked how often they experienced these issues: enjoyment or disinterest in engaging in activities previously enjoyed, how often they felt depressed, lethargic, and hopeless, and how much they experienced too much sleep, anxiety, or dreaming sleep. They were also asked how many times they felt like losing energy to work or were tired, how many times they ate a lot or experienced increased or lost appetite, how many times they felt causing harm to family or self, felt like a failure, self-blame and frustrated. The participants were also asked how many times they experienced difficulty with concentrating in activities like watching television and reading, and how many times they had these issues: people recognizing they are moving or talking slowly and vice versa and became moved, anxious and irritable more than usual, and how many times they felt hurting themselves or dying would be a better option. Other questions asked were about eye discomfort, tearing, and dryness. 

IBM SPSS was used to perform a statistical analysis and a chi-square test was used to attain a p-value between categorical independent and dependent data to determine the relationship. 

## Results

In this study, 401 participants responded to our questionnaire. However, after eliminating 12 participants who said no to being part of the study, the number dropped to 389 participants. Of these, 52.4% were male, and 44.6% were female. Moreover, less than 18-year-olds were 4%, 18-45 were 77.3%, 45-55 were 10.2%, and over 50 years were 5.5%. Further, 69.1% of them were single, and 25.7% were married. On education, 20.0% reached high school and below, 69.3% had completed a bachelor's or diploma, and 7.7% had a master's or doctorate degree. On employment, 43.6% were unemployed, 47.4% were employed, and 6.0% had retired. Of these, 60.3% confirmed having not been previously diagnosed with dry eye while 36.7% had been diagnosed with dry eye. (Table 1)

**Table 1:** Baseline characteristics of the study sample (n = 401).

Variables	Frequency	Percent
Would you like to participate in this study?		
yes	389	97.0
No	12	3.0
Sex		
Male	210	52.4
Female	179	44.6
Age group (years)		
< 18	16	4.0
18-45	310	77.3
45-55	41	10.2
≥50	22	5.5
Marital status		
Single	277	69.1
Married	103	25.7
Other	9	2.2
Education		
High school or below	80	20.0
Bachelor's or diploma	278	69.3
Master or doctorate	31	7.7
Employment status		
Unemployed	175	43.6
Employed	190	47.4
Retired	24	6.0
Previous diagnosis of dry eye		
No	242	60.3
Yes	147	36.7

In Table 2, on the part of medical history, 81% of the participants had no medical issues concerning physical ailments, while 13.2 % had suffered from other eye diseases. Concerning whether a participant had a refractive error, 32.7% of the participants had myopia, 8.0% had hyperopia, 13.2% had astigmatism, and 43.1% had no refractive error. On whether participants suffered from other eye diseases, 83.8% said no and 13.2% said yes. With respect to the feeling of the presence of a foreign body in their eyes, 36.4% of the participants mentioned "sometimes", 29.9% mentioned "scarcely", 16.7% mentioned "never", 10.5% mentioned "in most cases", and 3.5% mentioned "always". On experiencing eye redness, 32.9% confirmed they sometimes experienced it, 29.4% scarcely, 15.2% never, 16.0% in most cases, and 3.5% always. A total of 26.7% of the participants confirmed sometimes being sensitive to light, 26.4% being scarcely sensitive to light, 24.9% being never sensitive to light, 14.7% mentioned being sensitive to light in many cases, and 4.2% were always sensitive to light. Further, the research showed that 23.7% were diagnosed with anxiety, stress, and depression.

**Table 2:** Do you suffer from refractive error? And do you suffer from chronic disease? (n = 401).

Variables	Frequency	Percent
Do you suffer from refractive defects?		
Nearsighted	131	32.7
The length of view	32	8.0
Astigmatism	53	13.2
There is no	173	43.1
Do you suffer from other eye diseases?		
yes	53	13.2
no	336	83.8
Do you suffer from physical ailments?		
yes	64	16.0
no	325	81.0
Have you ever felt the presence of a foreign body in your eyes?		
Sometimes	146	36.4
Scarcely	120	29.9
Never	67	16.7
In many cases	42	10.5
Always	14	3.5
Have you ever suffered from redness in the eyes?		
Sometimes	132	32.9
Scarcely	118	29.4
Never	61	15.2
In many cases	64	16.0
Always	14	3.5
How often have your eyes been sensitive to light?		
Sometimes	107	26.7
Scarcely	106	26.4
Never	100	24.9
In many cases	59	14.7
Always	17	4.2
Have you been diagnosed with depression, stress or anxiety?		
No	294	73.3
Yes	95	23.7

As presented in Table 3, participants were required to identify whether they had experienced the following problems: They were asked if they enjoyed or lacked interest in participating in things they previously enjoyed doing, and 416% of the participants enjoyed and were disinterested some days, 23.4% enjoyed and were disinterested most days, 25.2% had no problem at all, and 6.7% were disinterested almost every day. A total of 42.9% felt depressed, lethargic, and hopeless some days only, 21.4% on most days, 21.7% not at all, and 11.0% felt so almost every day. On sleeping too much, dreaming anxiety, or sleep, 38.2% had that experience some days, 24.2% on most days, 20.0% not at all, and 14.7% almost every day. On losing energy or feeling tired to work, 36.4% felt so some days, 22.2% on most days, 26.7% not at all, and 11.7% almost every day. Further, 27.4% felt they could harm their family or themselves, felt like failures, were frustrated and blamed themselves some days, 21.9% felt this way on most days, 37.9% not at all, and 9.7% almost every day. A total of 36.2% of participants experienced difficulties in concentrating and doing tasks some days, 19.7% felt this way on most days, 32.7% not at all, and 8.5% almost every day. On how many times the participant felt anxious, moved or irritated when others noticed they were moving or talking slowly, 26.2% experienced that some days, 13,2% on most days, 49.9% not at all, and 7.7% almost every day. And lastly, when asked about how many times they felt hurting themselves or dying would be better, 22.7% answered some days, 8.2% answered most days, 62.3% answered not at all, and 3.7% answered almost every day. 

**Table 3:** How many times have you felt any of the following problems:(n = 401).

Variables	Frequency			Percent
How often have you experienced any of the following problems: [lack of interest or enjoyment in doing things that you previously cared for or enjoyed]?				
Some days only		167	41.6	
Most days		94	23.4	
Not at all		101	25.2	
Almost every day		27	6.7	
How many times have you felt any of the following problems: [feeling hopeless, lethargic, or depressed]?				
Some days only		172	42.9	
Most days		86	21.4	
Not at all		87	21.7	
Almost every day		44	11.0	
How many times have you experienced any of the following problems: [dreaming sleep or anxiety, or sleeping too much]?				
Some days only		153	38.2	
Most days		97	24.2	
Not at all		80	20.0	
Almost every day		59	14.7	
How many times have you felt any of the following problems: [feeling tired or losing energy to do work]?				
Some days only		159	39.7	
Most days		115	28.7	
Not at all		62	15.5	
Almost every day		53	13.2	
How many times have you felt any of the following problems: [Loss of appetite or increase and eating a lot]?				
Some days only		146	36.4	
Most days		89	22.2	
Not at all		107	26.7	
Almost every day		47	11.7	
How many times have you felt any of the following problems: [Feeling frustrated, self-blame, feeling like a failure, or causing harm to yourself and your family]?				
Some days only		110	27.4	
Most days		88	21.9	
Not at all		152	37.9	
Almost every day		39	9.7	
How many times have you felt any of the following problems: [difficulty concentrating on doing things such as: (reading, watching TV,…)]?				
Some days only		145	36.2	
Most days		79	19.7	
Not at all		131	32.7	
Almost every day		34	8.5	
How many times have you had any of the following problems: [Have people noticed you talking or moving slowly, or vice versa - that you became irritable and anxious and moved more than usual]?				
Some days only		105	26.2	
Most days		53	13.2	
Not at all		200	49.9	
Almost every day		31	7.7	
How many times have you felt any of the following problems: [Have you thought dying, or hurting yourself, would make you better]?				
Some days only		91	22.7	
Most days		33	8.2	
Not at all		250	62.3	
Almost every day		15	3.7	

## Discussion

This study aimed to investigate the connection between depression and dry eye disease. To the best of our knowledge, this is the first study to determine a correlation between these two conditions. In our study, we investigated 401 Saudi Arabians from the Aseer region. We utilized a three-sectioned questionnaire as shown in Tables 1-3 to determine the stress, anxiety and depression, and prevalence of dry eye disease as well as mood changes. 

A total of 36.7% of the participants had suffered from dry eye disease and 23.7% were diagnosed with anxiety, stress, and depression. The results also indicate that a good number of the participants had symptoms of dry eye wherein 92.7% of the participants at some point experienced light sensitivity, 93.5% experienced eye redness at some point, and 93.5% experienced a foreign object in their eyes at some instance. Mood changes due to dry eye or anxiety, stress, or depression have been realized. For example, 34.6% of the participants thought of harming themselves, or that dying is a better solution, and 47.1% felt uncomfortable with the way others viewed them. Numerous studies have been conducted to understand the association between depression and dry eye disease [7, 8]. However, to our knowledge, no prior study has been conducted using this questionnaire to determine the relationship between depression and DED.

The results of this study signify the importance of detecting depression among individuals with dry eye disease. They also identify the role dry eye disease or depression symptoms play in the development of those diseases. The results of this study are in line with previous research [7-13]

Further, the study found a direct impact of DED (dry eye disease) and depression on the daily activity of people wherein 81.6% of the participants would feel tired or like they are losing energy for work at some point, and 77.1% had sleeping issues [13]. Other emotions they experienced are anxiety, frustration, self-blame, feelings of failure, or causing harm to self and family [11]. Depression and anxiety are seen to affect an individual’s mental state. Further, the results of this study have practical implications for the country's Ministry of Health, society, and psychologists. There is a need to make the general population aware of the severity of eye disease and its connection to depression.

Limitations of the study

This study has some limitations that further future research can cover. First, the study collected data from a specific part of Saudi Arabia. In the future, research can select another area or country. Secondly, the sample size of this study consisted of 401 participants. Future research can increase the data set to cover more details on this topic. Finally, future researchers can also select other variables likely to cause depression.

## Conclusions

In Saudi Arabia, there is a high prevalence of dry eye disease, stress, anxiety, and depression. This study is significant for the Ministry of Health and Society to curb this rising issue. After analyzing the connection between dry eye disease and depression, our study concludes that dry eye disease is related to depression. Depression is significantly prevailing among females and youth, more than among older people. This study recommends that with the help of primary care clinics and ophthalmology, the psychological well-being of the affected can be improved, thus improving the quality of life.
